# Plasmonic Surface of Metallic Gold and Silver Nanoparticles Induced Fluorescence Quenching of Meso-Terakis (4-Sulfonatophenyl) Porphyrin (TPPS) and Theoretical–Experimental Comparable

**DOI:** 10.1007/s10895-022-03022-0

**Published:** 2022-08-31

**Authors:** Ahmed A. Aboalhassan, Samy A. El-Daly, El-Zeiny M. Ebeid, Mahmoud A. S. Sakr

**Affiliations:** 1 Chemistry Department, Faculty of Science, Tanta University, Tanta, Egypt; 2 Chemistry Department, Center of Basic Science, Misr University for Science and Technology (MUST), 6th October City, Egypt

**Keywords:** Gold and silver nanoparticles, Porphyrin derivatives, Fluorescence quenching, Non-linear stern–volmer plots, DFT

## Abstract

Colloidal metallic nanoparticles have attracted a lot of interest in the last two decades owing to their simple synthesis and fascinating optical properties. In this manuscript, a study of the effect of both gold nanoparticles (Au NPs) and silver nanoparticles (Ag NPs) on the fluorescence emission (FE) of TPPS has been investigated utilizing steady-state fluorescence spectroscopy and UV–Vis spectrophotometry. From the observed electronic absorption spectra, there is no evidence of the ground state interaction between metallic Au NPs or Ag NPs with TPPS**.** On the other side**,** the FE spectra of TPPS have been quenched by both Ag and Au NPs. Via applying quenching calculations, Ag NPs showed only traditional static fluorescence quenching of TPPS with linear Stern–Volmer (SV) plots. On the contrary, quenching of TPPS emission by Au NPs shows composed models. One model is the sphere of action static quenching model that prevails at high quencher concentrations leading to non-linear SV plots with positive deviation. However, at low Au NPs concentrations, traditional dynamic quenching occurs with linear SV plots. The quantum calculations for TPPS structure have been obtained using Gaussian 09 software: in which the TPPS optimized molecular structure was achieved using DFT/B3LYP/6-311G (d) in a gaseous state. Also, the calculated electronic absorption spectra for the same molecule in water as a solvent are obtained using TD/M06/6-311G +  + (2d, 2p). Furthermore, the theoretical and experimental results comparable to UV–Vis spectra have been investigated.

## Introduction

Porphyrins (PPs) are largely colored heterocyclic macrocycle organic compounds with their main absorption bands characterized via very large molar absorptivity. The intense *B-band* or “*Soret band*” found around 400 nm is characteristic of macrocyclic conjugation. Additionally, in the Soret band, there are between two and four weaker bands called “*Q-bands*” situated between 480–700 nm [1]. The number and intensity of those bands can give information on the substitution pattern of the PP and whether it is mutilated or not. Metal-free PPs have a Soret band and four Q-bands, whereas, metal-PPs generally have a Soret band and two Q-bands [2]. The significance in the photophysical characterizations of PP and PP-like geometrical structures has enhanced significantly during the last decades owing to the long-range variety of their applications. For instance, PPs are utilized for the enhancement of nonlinear (NL) photonic devices, like optical limiters [3–5] and optical switches [6, 7]. Their high optical (NL) arise from their geometrical structures existing extended π-conjugated systems [8, 9]. Water-soluble PPs and metalloporphyrin have a lot of potential applications in bioscience [10–13]), and material science [14–16]*.* Also, in medicine, water-soluble PPs investigated anti-HIV [17] and antibacterial activity [18], and it was utilized as active compounds for singlet oxygen imaging of single cells [19] and singlet oxygen photosensitization in skin fibroblasts [20]. One of the most significant water-soluble PP derivatives is the synthetic meso-tetrakis (4-sulfonatophenyl) porphyrin (TPPS) [10].

Meso-tetrakis (4-sulfonatophenyl) porphyrin or (5, 10, 15, 20-tetrakis (4-sulfonatophenyl) porphyrin, abbreviated as (H_2_TPPS^− 4^) or (TPPS)**,** is a substituted PP-type molecule with four meso-phenyl rings containing SO_3_^−^ groups in the *para-* positions which make this PP derivative a very good example of anionic water-soluble PP derivatives [21]. TPPS has attracted a lot of researchers' interests and has been studied as a promising sensitizer for PDT [22]. Besides this TPPS has NL optical absorption [23], which can lead to its application in photonic devices such as optical limiters [24]and switches [25].

Rahman and Harmon investigated absorbance changes and static quenching of fluorescence of TPPS utilizing trinitrotoluene (TNT) [26]. Also, Kathiravan et al*.* investigated the fluorescence quenching (FQ) of TPPS via certain pyrimidines *using* steady-state and time-resolved techniques [27]. They found that the Q processes obey the SV equation with linear plots. The FQ of TPPS by applying colloidal nano TiO_2_ [28], and colloidal nano CdS [29] was reported. It was found that TPPS was adsorbed on both colloidal semiconducting nanomaterials (TiO_2_ and CdS) surfaces through the sulfonate SO_3_^**−**^ group as an anchoring group [29]. Kathiravan et al*.* reported the FQ of TPPS with colloidal metal-semiconducting (Au/TiO_2_, and Ag/TiO_2_) core–shell nanomaterials [29]. It was found that TPPS showed higher rates in cases of metal–semiconductor nanomaterials (Au/TiO_2_, and Ag/TiO_2_) compared with electron transfer to colloidal TiO_2_.

The present manuscripts aimed the investigation the interaction of TPPS with Au NPs and Ag NPs using electronic absorption and fluorescence techniques. The second-order rate constants of fluorescence quenching of TPPS in viscous and non-viscous media were also determined. Also, the theoretical–experimental comparable for UV-spectra and the accurate functional and basis set for TPPS geometrical structure have been investigated.

## Experimental Details

### Materials

The sodium salt of **TPPS**, tetrachloroauric acid (99.9%, HAuCl_4_.3H_2_O), and silver nitrate (AgNO_3_) were obtained from *Sigma-Aldrich*. Citrate trisodium salt (95%, C_6_H_5_O_7_Na_3_.2H_2_O), hydrochloric and nitric acids were purchased from *Fluka*.
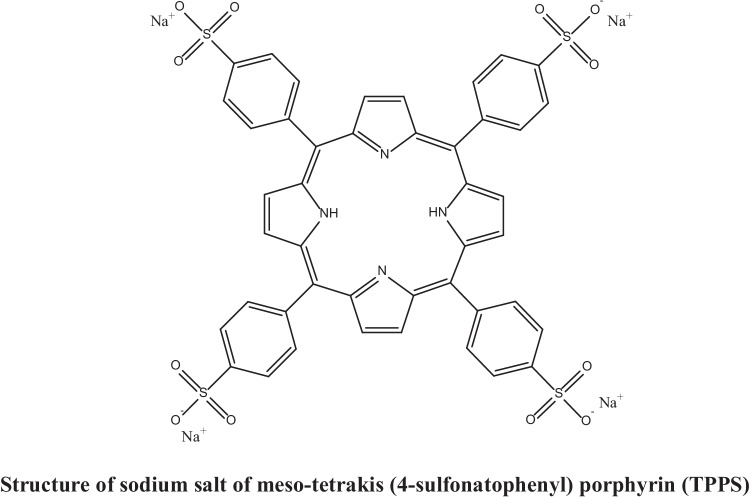


### Synthesis of Metallic Nanoparticles

#### Synthesis of Gold NPs

Approximately 13 nm diameter Au NPs were prepared via utilizing the citrate reduction of HAuCl_4_.3H_2_O [30, 31]. An aqueous solution of HAuCl_4_.3H_2_O (1 mM, 100 mL) was brought to reflux while stirring, and then 10 mL of a 1% trisodium citrate solution (as nucleating and reducing agent) was added quickly, which resulted in a change in solution color from pale yellow to deep red. After the color change, the solution was refluxed for an additional 15 min and allowed to cool to room temperature. A typical solution of 13 nm diameter gold particles exhibited a characteristic surface plasmon band around 520 nm. The size and mono-dispersity of the resulting NPs were well documented for this method of synthesis [32]. The mechanism of the successive reduction of [AuCl_4_] ^**−**^ ions into metallic Au NPs is explained in detail in the literature [33]. Form the electronic absorption spectra of the prepared 13-nm diameter of 2.01 nM concentration and 0.3302 optical density (OD) at λ_max_ = 520 nm, the extinction coefficient **ε** (λ) of the prepared Au NPs was calculated as 1.6 × 10^8^ M^−1^ cm^−1^ comparable literature value of 8 × 10^8^ M^−1^ cm^−1^ and 2.4 × 10^8^ M^−1^ cm^−1^ [34].

#### Synthesis of Silver NPs

Ag NPs were prepared by applying the citrate reduction of AgNO_3_ [32]. An aqueous solution of AgNO_3_ (1 mM, 125 ml) was heated until it starts to boil, and then 5 ml of a 1% trisodium citrate solution (as nucleating and reducing agent) was added quickly, which resulted in a change in solution color to pale yellow. After the color changed, the solution was removed from the heating element, and allowed to stir until cool to room temperature. A typical solution of silver nanoparticles exhibiting a characteristic surface plasmon band around 420 nm was obtained [32]. For 10 nm diameter Ag NPs, the extinction coefficient was calculated as 1.328 $\times$ 10^7^ M^−1^ cm^−1^ compared with the literature value [35]. The prepared Au and Ag NPs were characterized by electronic absorption spectroscopy and by Transmission Electron Microscopy (TEM).

### Spectroscopic Measurements and Nanoparticle Characterizations

The electronic absorption spectra had been recorded utilizing the Shimadzu UV-3101 PC spectrophotometer. The steady-state fluorescence spectra had been recorded employing the Perkin-Elmer LS-50B scanning Spectrofluorometer, utilizing matched quartz cuvettes. The nanoparticle size changed into characterized with the aid of using a transmission electron microscope (TEM), JEOL JEM-100SX Electron Microscope with a field gun, and an accelerating voltage of 80 kV.

### DFT and TD-DFT Calculations

The optimized MSs for TPPS in the gaseous state are obtained using the DFT/B3LYP/6-311G (d) method [36–38]. The UV–Vis absorption spectra for TPPS and in H_2_O are calculated via applying TD/M06/6-311G +  + (2d, 2p).

## Results and Discussion

### Electronic Absorption Spectra

**Fig. 1:**
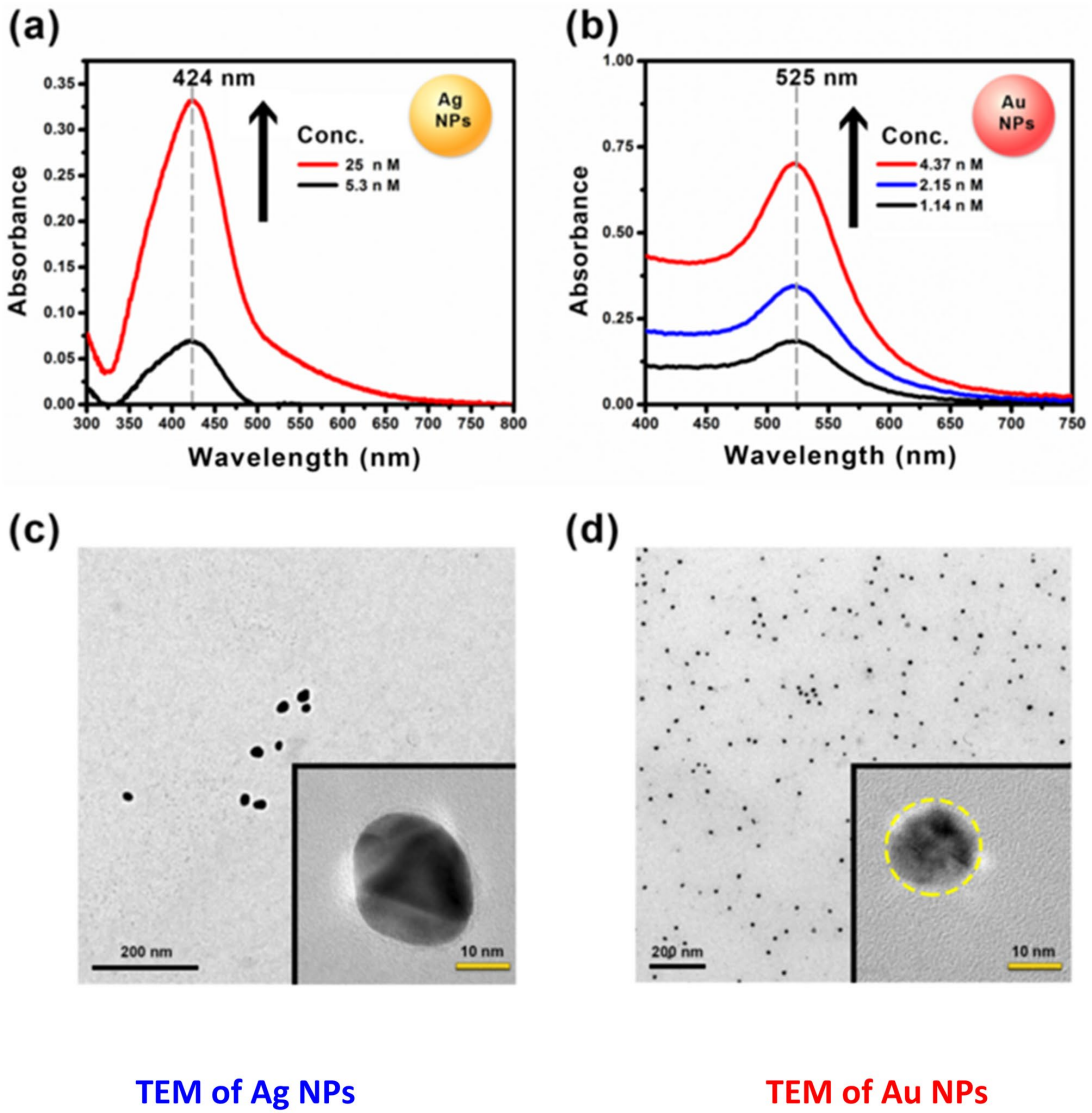
Electronic absorption spectra of Ag NPs (5.3 nM and 25 nM) (**a**) and Au NPs (1.14 nM, 2.0 = 15 nM and 4.37 nM) (**b**). TEM of Ag NPs (**c**) and Au NPs (**d**)

The electronic absorption spectra of 5.03 and 25.0 nM of Ag NPs and those of 1.14, 2.15, and 4.37 nM of Au NPs were collected in Fig. 1(a and b) with the same spectral features as reports [39]. As presented in Fig. 1a and b), the maximum absorbance of Ag and Au NPs is increased with increasing its concentrations. The object is to display the ELECTRONIC ABSORPTION SPECTRA of different concentrations used in quenching of TPPS geometrical structure (GS). Figure 1 (c and d) show the TEM micrographs of the synthesized Au NPs and Ag NPs. It confirms the nanoscale dimension of the NPs and shows their average diameters of about 13 and 10 nm, respectively.

**Fig. 2:**
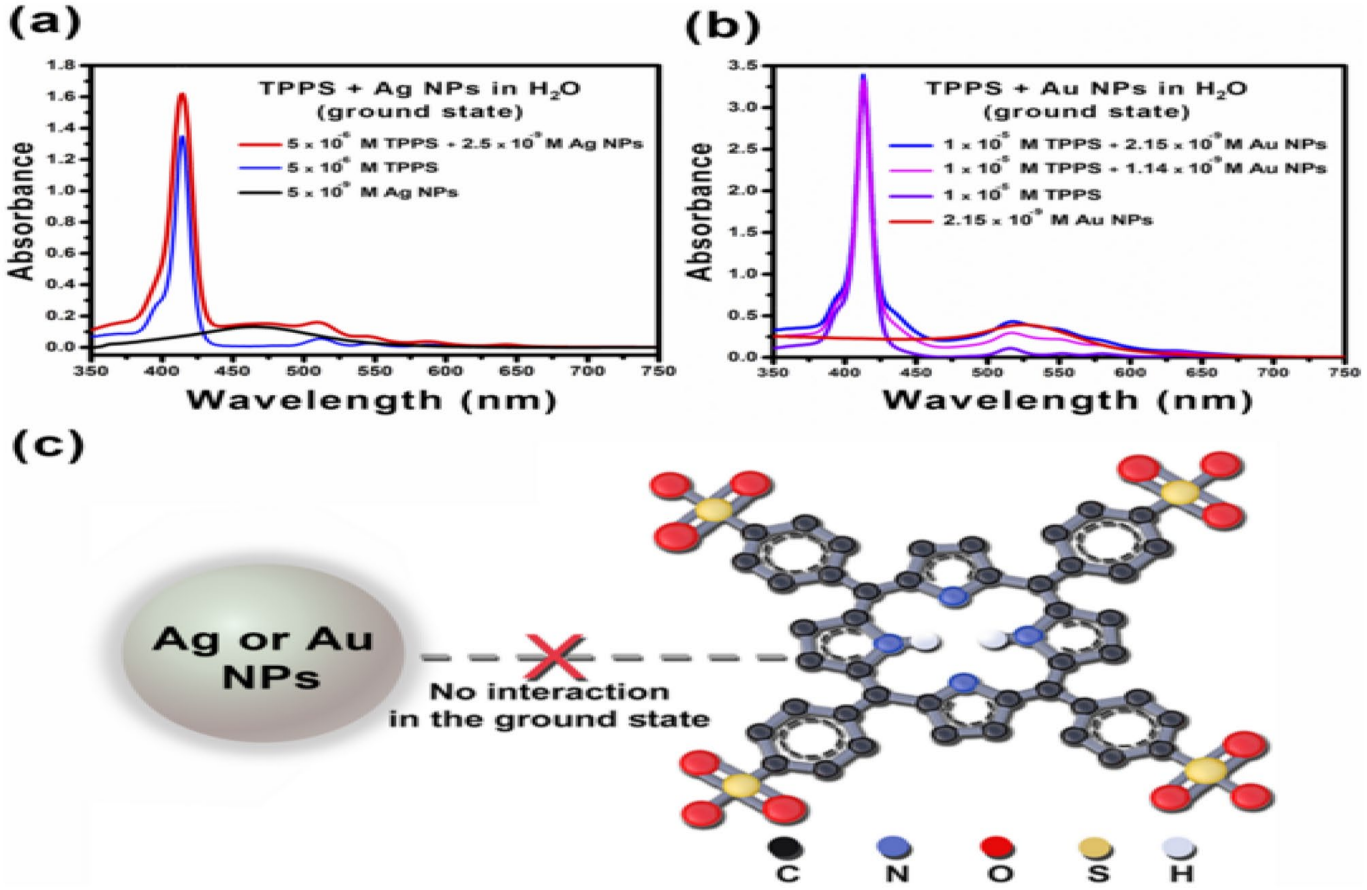
Electronic absorption spectra of TPPS of 5 × 10^–6^ (**a**) and 1 × 10^–5^ (**b**) M TPPS, TPPS/ 2.5 nM of Ag NPs (**a**) and TPPS/ various concentrations of Au NPs (1.14 and 2.15 nM) (**b**). The interaction between TPPS and Ag or Au NPs in the ground state (**c**)

The electronic absorption spectra of 5 × 10^–6^ M aqueous solution of TPPS GS were recorded in the absence and presence of 2.5 and n M aqueous Ag NPs as shown in Fig. 2a. We noted the shape and band maxima of absorption spectra remain unchanged upon increasing the concentration of Ag NPs. Also, these behaviors are observed with Au NPs as presented in Fig. 2b. Those referring to there is no observable interaction or photochemical reaction between TPPS and Ag or Au NPs in the ground state under the prevailing experimental conditions (see Fig. 2c). reported similar behavior because of Hematite NPs on the spectra of one of the coumarin dyes.

### Fluorescence Quenching

#### Effect of Ag NPs on Fluorescence Emission of TPPS

**Fig. 3:**
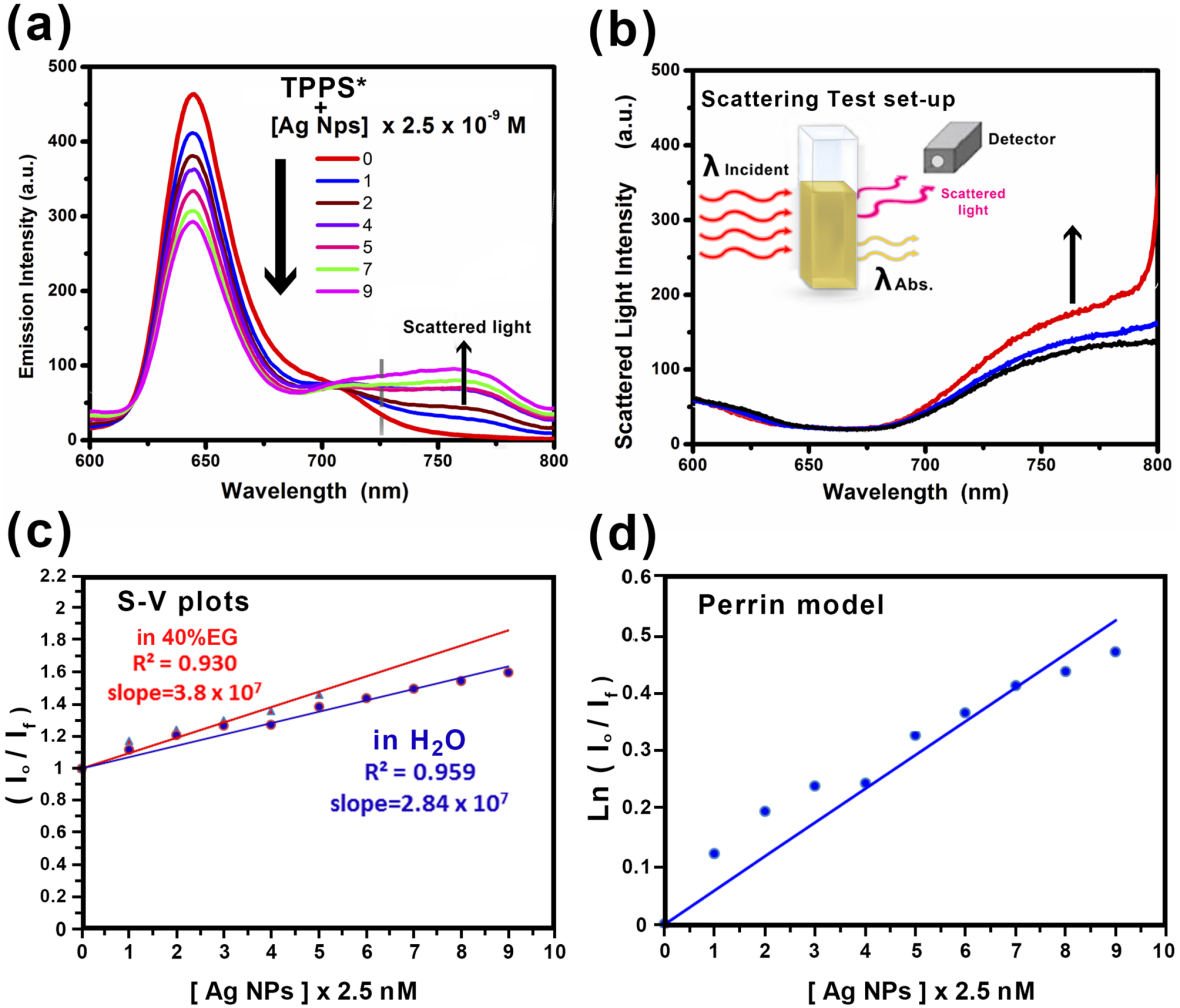
Emission spectra of 1 × 10^–5^ M of (TPPS) in water (λ_ex_ = 414 nm) in the absence and presence of Ag NPs (**a**). Spectra of scattered light intensity (**b**). Stern–Volmer (S-V) (**c**) and Perrin model (**d**) plots for quenching of 1 × 10^–5^ M TPPS by Ag NPs

The fluorescence emission of TPPS was investigated in the presence of variable concentrations of Ag NPs as a quencher in two different media; one of them contains only pure water and the other contains 40% ethylene glycol (EG) by volume in water at room temperature. Figure 3a shows the fluorescence emission spectra of 1 × 10^–5^ M TPPS in the presence of variable concentrations of Ag NPs in pure water. The fluorescence emission of TPPS exhibits a maximum at 650 nm upon excitation at 414 nm. As the concentration of quencher was increased, λ_max_ of the fluorescence emission bands at 650 nm decreased while the fluorescence emission intensity of the emission band at 760 slightly increases. Under the present experimental conditions, no such quenching of TPPS was seen in the presence of the low concentrations of capping agent indicating that Ag NPs are responsible for the fluorescence quenching. The fluorescence emission band appearing beyond 760 nm is assigned to light scattering from colloidal Ag NPs as shown in Fig. 3a. Long-wavelength light scattering from colloidal Ag NPs was reported earlier by Klitgaardet al*.* [40]. This scattering was explained by a theory based on interference between two surface plasmon resonances of higher concentrations of colloidal nanoparticles [40]. We measured this scattering from the prepared Ag NPs under the prevailing experimental conditions as shown in Fig. 3b.

Figure 3c shows the Stern–Volmer (S-V) plot derived from Eq. (1) of fluorescence emission quenching of TPPS by Ag NPs as a quencher [41].

1 $$\frac{{I}_{o}}{I} =1+ {K}_{sv }[Q]$$

where I_o_ and I are the fluorescence emission intensities in the absence and presence of the quencher concentration $\left[Q\right]$, respectively. The ${K}_{sv}$ was calculated as 2.84 × 10^7^ M^−1^ and 3.8 × 10^7^ M^−1^ in pure water and 40% EG/water, respectively. The quenching efficiency increases as the medium viscosity increases indicating that the quenching process is not completely diffusion–controlled. This is consistent with a static quenching model in which increasing the medium viscosity leads to a cage effect that enhances the fluorophore uptake on Ag NPs surfaces. Taking the fluorescence lifetime of TPPS in the absence of Ag NPs as 10.4 ns [42], the values of k_q_ = K_sv_ /τ are calculated as 2.73 × 10^15^ M^−1^ s^−1^ in water. This value is much higher than the diffusion rate constant k_d_ (k_d_ = 1.095 × 10^9^ M^−1^ s^−1^) for water. This suggests that the static quenching mechanism plays a major role in the quenching of TPPS by Ag NPs.

The Perrin model was valid for the quenching process of TPPS by Ag NPs. The Perrin relationship [43] is given by Eq. (2):

2 $$In\left(\frac{I_o}I\right)=VN_o\left[Q\right]\;\;\;;\;\;V=\frac43\pi r^3$$

where *I*_*o*_ and *I* are fluorescence emission intensities in the absence and presence of a quencher, *V* is the volume of the quenching sphere in cubic centimeters, *N*_*o*_ is the Avogadro's number, [*Q*] is the molar concentration of the quencher, and *r* is the radius of quenching sphere volume. Figure 3d shows a linear plot ln(*I*_*o*_* /I*) versus [*Q*] with a slope equal to *VN*_o_. Accordingly, the volume and radius of the quenching sphere were 3.85 × 10^–14^ cm^3^ and 209.5 nm, respectively.

#### Effect of Au NPs on Fluorescence Emission of TPPS

**Fig. 4:**
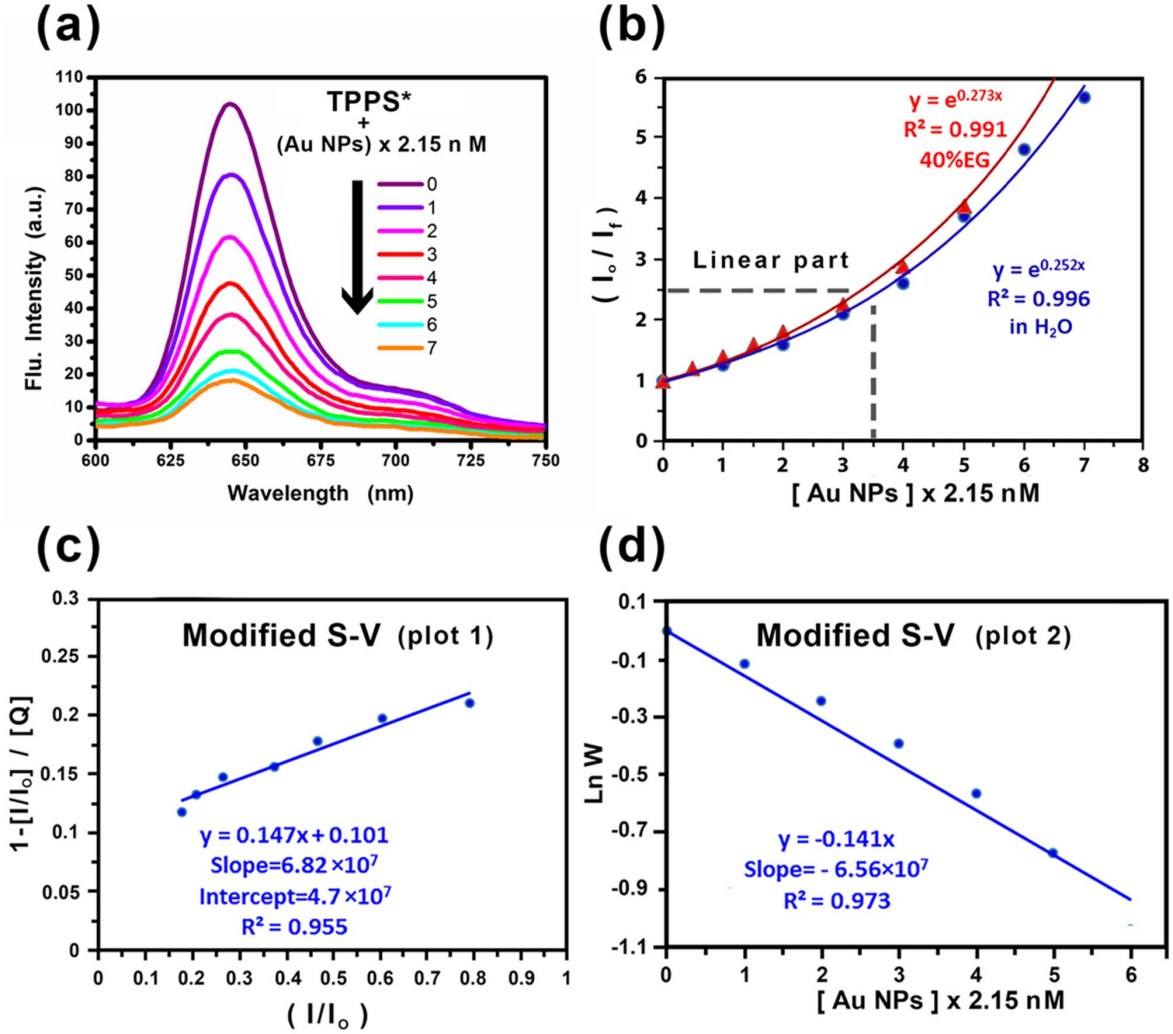
Emission spectra of 1 × 10^–5^ M of (TPPS) in water (λ_ex_ = 414 nm) in the absence and presence of Au NPs (**a**). Stern–Volmer (S—V) (**b)** and modified (S – V) (**c** and **d**) plots for quenching of 1 × 10^–5^ M TPPS by Ag NPs

The fluorescence emission of TPPS was studied in the presence of variable concentrations of Au NPs as a quencher in two different media containing 0% and 40% EG/water by volume in H_2_O at room temperature. When the concentration of Au NPs was increased gradually, the fluorescence emission intensity decreased significantly as a consequence of TPPS fluorescence quenching without any appreciable change in position and shape of the emission band, Fig. 4a. This indicates the absence of molecular aggregation under the prevailing experimental conditions.

Upward curvatures were observed, as a result of the quenching process of TPPS by Au NPs in H_2_O and 40% EG solutions, by applying the traditional SV equation (Eq. 1), Fig. 4b. Each plot shows two trends, namely the linear and nonlinear portions. From the linear portions of the SV plots, the values of *K*_***SV***_ in water and 40% EG solutions were calculated as1.73 × 10^8^ and 2.04 × 10^8^ M^−1^, respectively. The bimolecular quenching rate constant (***k***_***q***_) has been calculated as *k*_*q*_ = 1.663 × 10^16^ M^−1^ s^**−1**^ (where, K_SV_ = *k*_*q*_ τ_o_; taking lifetime of TPPS as τ_o_ = 10.4 ns in water [43]. This value is much higher than the diffusion rate constant in water **(***k*_*d*_ = 1.095 × 10^9^ M^−1^ s^−1^**)** at room temperature.

**Fig. 5:**
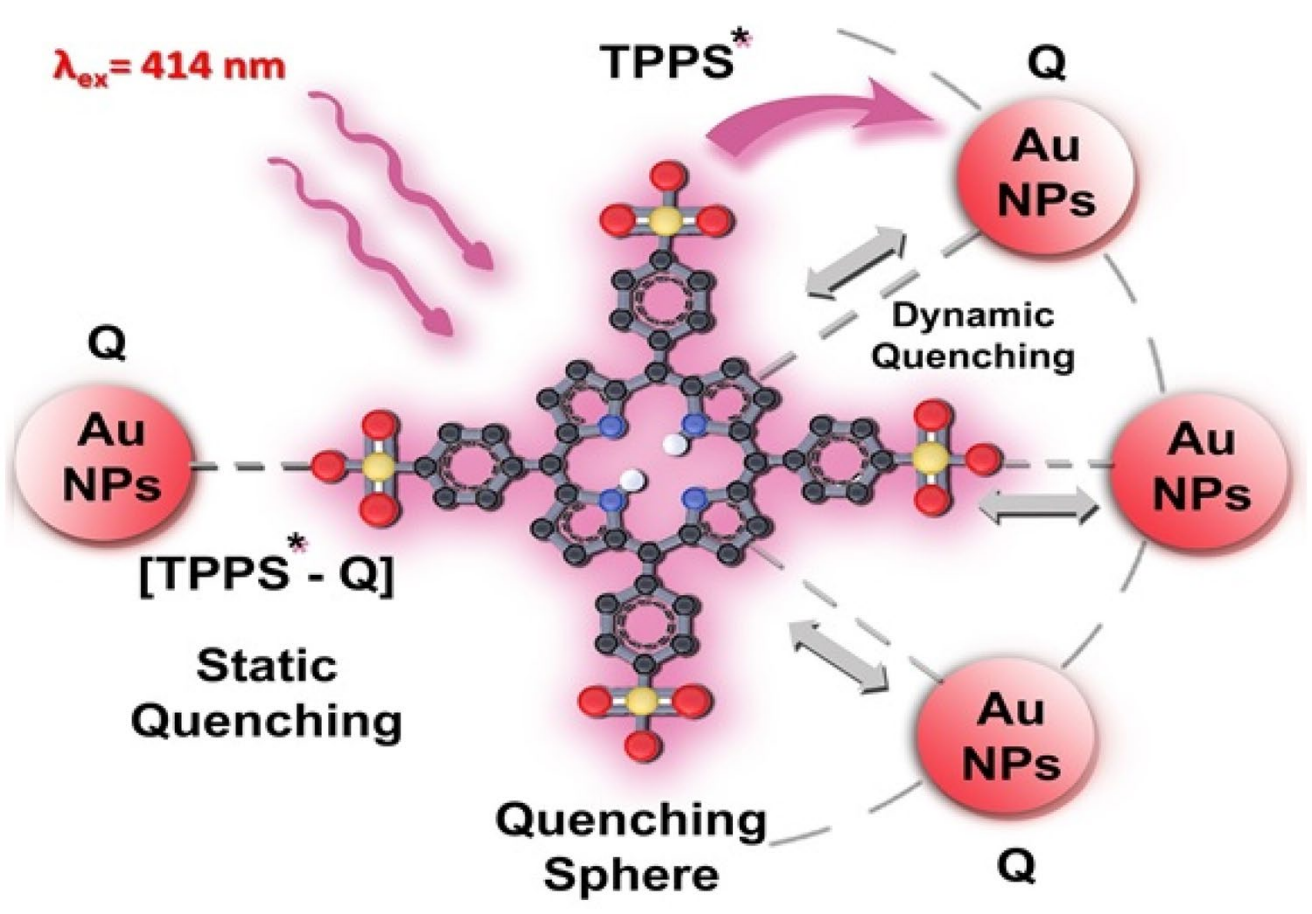
Schematic illustration of the interaction mechanism of TPPS* + Au NPs

The nonlinear plots showed positive deviation and similar behaviors were observed earlier [44]. The positive deviation from linearity suggests that the quenching is not purely static or dynamic and may be due to simultaneous dynamic and static quenching mechanisms as shown in Fig. 5. The analysis of data was carried out by employing the sphere of action static model using the modified form of SV equation [44] given as:

3 $$\left(\frac{{\mathrm{I}}_{\mathrm{o}}}{\mathrm{I}}\right)=\frac{1}{\mathrm{W}}(1+{K}_{sv} [Q])$$

The additional factor W is expressed as:

4 $$W={e}^{- {\varvec{V}}\boldsymbol{ }[Q]}$$

where *‘*V*’* is the static quenching constant that represents an active volume element surrounding the fluorophore in its excited state. As W depends on the quencher concentration [Q], the SV plots for a quencher with a high quenching ability generally deviate from linearity. Thus, it is worth rewriting Eq. (3) as:

5 $$\frac{1- ( \frac{I}{{ I}_{o}})}{[Q]}={K}_{sv}( \frac{I}{{ I}_{o}})+\frac{1-W}{[Q]}$$

According to Eq. (5), $\frac{1- ( \frac{\mathrm{I}}{{\mathrm{ I}}_{\mathrm{o}}} )}{[\mathrm{Q}]}$ was plotted against $( \frac{{\mathrm{I}}_{\mathrm{o}}}{\mathrm{I}} )$ as shown in Fig. 4c. The SV dynamic quenching constant (K_SV_ = 6.82 × 10^7^), was obtained by the least-square fit method determining the slope. The intercept of the plot was used to calculate *W* values for each quencher concentration [*Q*]. Plotting of *lnW* against [*Q*] gave a linear correlation with a negative slope that equals the static quenching constant (*V* = -6.56 × 10^7^), as in Eq. (4) as illustrated in Fig. 4d. From the value of *V* one can calculate *r.*

The calculated *K*_*SV,*_* V,* and *r* values are listed in Table 1. The radii of the fluorophore (R_Y_) and the quencher (R_Q_) molecules were determined by using the additive model [45]. *R* is the sum of both molecular radii is named encounter distance *R*. As seen, the value of kinetic distance *r* was greater than the encounter distance *R.* Therefore, the static effect takes place irrespective of the ground state complex formation provided the reactions are limited by diffusion indicating that the sphere of action model holds well [45]. Further, it may also be noted that a positive deviation in the SV plot is expected when both static and dynamic quenching occur simultaneously [45].

**Table 1:** Quenching parameters of TPPS—Au NPs system in H_2_O

*K*_*SV*_ (M^−1^)	*V* (M^−1^)	*R*_*Y*_ (Å)	*R*_*Q*_ (Å)	*R* = *R*_*Y*_ + *R*_*Q*_ (Å)	*r* (*nm*)
6.82 × 10^7^	6.56** × **10^7^	8.2	65	73.2	2962

### Quantum Calculations

#### DFT Calculations

**Fig. 6:**
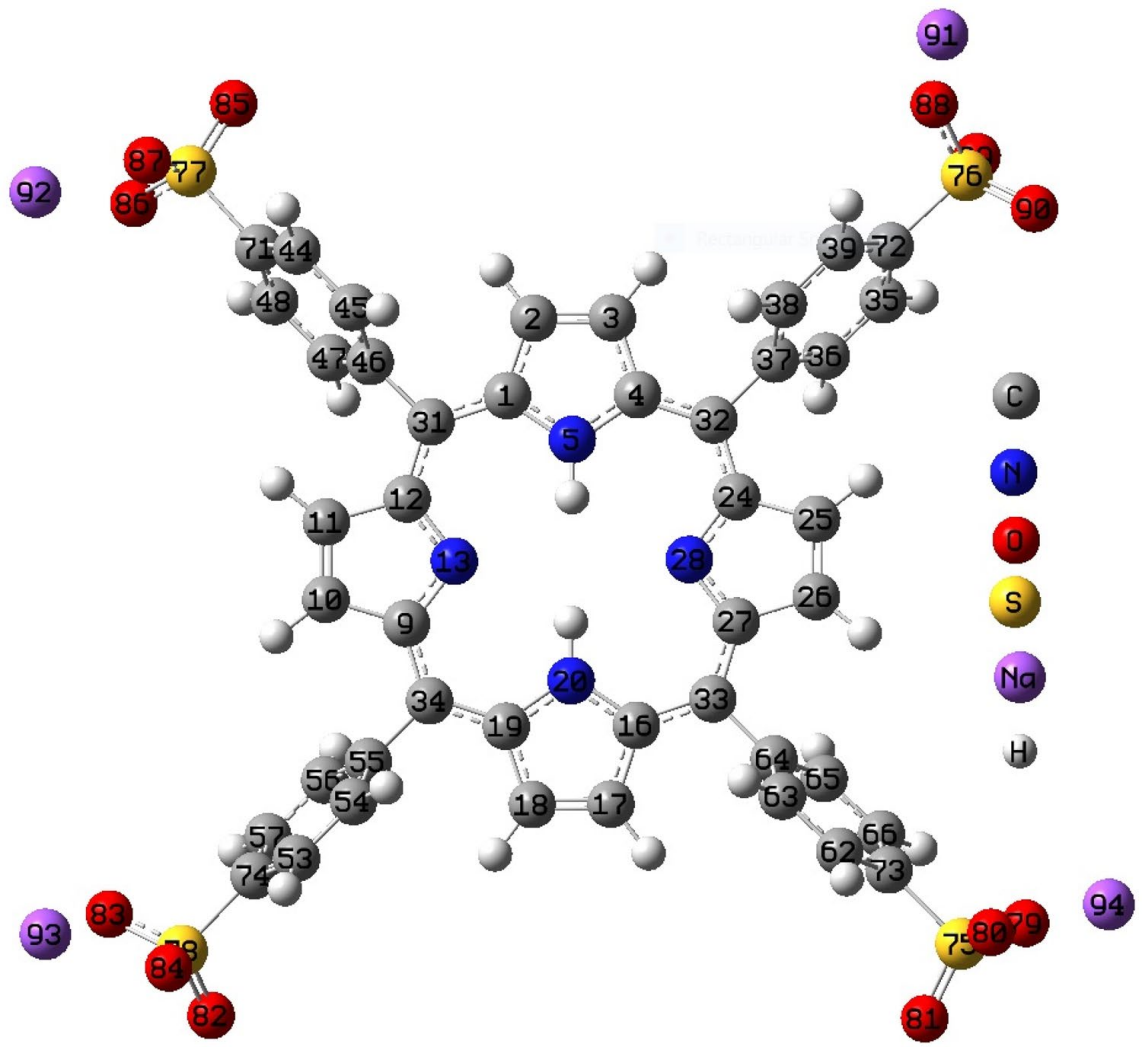
Optimized geometrical structure for TPPS in the ground state using DFT/B2LYP/6-311G(d) in the gaseous phase

Utilizing the DFT method, the electronic Geometrical structure (GS) for the ground state of TPPS was computed. The electronic GS optimization was investigated at the DFT/B3LYP/6–311G (d) method in a gaseous state; the result is obtained in Fig. 6. The labeled optimized GS for the TPPS compound is obtained in Fig. 6. The TPPS GS is not planar where the phenyl sodium sulfate rotates out the PP group in TPPS via 71.67° to prevent the steric hindrance as shown in Fig. 6. Selected optimized structural parameters (bond length in ˚A, bond angle and dihedral angle in degree (^o^) computed for TPPS in the ground (S_0_) and first excited states (S_1_) via applying DFT/B3LYP/6-311G(d) method in the gaseous state are presented in Table 2. DFT was used to get the ground state GS of TPPS in the gaseous state. Also, TD-DFT was applied to get an electronically excited state (S_1_) for TPPS in the gaseous phase. Various comments can be concluded from Table 2 for TPPS as follow; (1) Owing 71.67° (C45-C46-C31-C1) dihedral angle over the entire TPPS backbone of the molecule, the TPPS GS is not planar as shown in Fig. 6 (2) The difference between C–C single bonds and C = C double bonds are very small due to high conjugations. (3) The obtained bond angles refer to the sp^2^ hybridization over the entire TPPS backbone GS. (4) As presented in Table 2 and Fig. 6, the All-bond lengths, bond angles, and dihedral angles for TPPS GS are not affected upon excitation from S_o_ to S_1_. (5) Upon excitation from S_o_ to S_1_, the Mulliken charge of all atoms in TPPS GS is decreased except for the charge of C71, C46 and C2 atoms (See Table 2). (6) It is apparent from Table 2 that the terminal oxygen and sulfur atoms (i.e. (SO_3_^−^ group) in the S_o_ and S_1_ states have a large Mulliken charge compared to the nitrogen atoms in the middle ones. These findings could lead to the binding of the excited state near oxygen atoms in SO_3_^−^ to the Au or Ag NPs [46]. This is referring to a good agreement with the experimental results.

**Table 2:** Some important quantum parameters like bond length in (A), bond and dihedral angles in degree, and Mulliken charge for TPPS GS in the ground (S_o_) and first excited (S_1_) states

Designation	S_o_	S_1_	atom	Mulliken Charge	
				S_o_	S_1_
C44-C45	1.391	1.391	N5	-0.786	-0.182
C46-C31	1.498	1.498	N28	-0.794	-0.665
S77-O85	1.461	1.461	N20	-0.787	-0.175
C1-N5	1.374	1.374	N13	-0.793	-0.695
C1-C2	1.433	1.433	O85	-1.039	-0.717
C71-S77	1.801	1.801	O86	-1.093	-0.720
C48-C71-C44	120.71	120.71	O87	-1.091	-0.548
C45-C46-C47	118.6	118.6	S77	2.861	1.279
C1-C2-C3	108.16	108.16	C71	-0.343	-0.648
C31-C1-N5	126.68	126.68	C31	-0.101	-0.068
C45-C46-C31-C1	71.67	71.67	C46	-0.071	-0.377
C45-C46-C31-C12	108.58	108.58	C1	0.386	0.266
C46-C31-C1-N5	178.1	178.1	C2	-0.428	-0.584

**Fig. 7:**
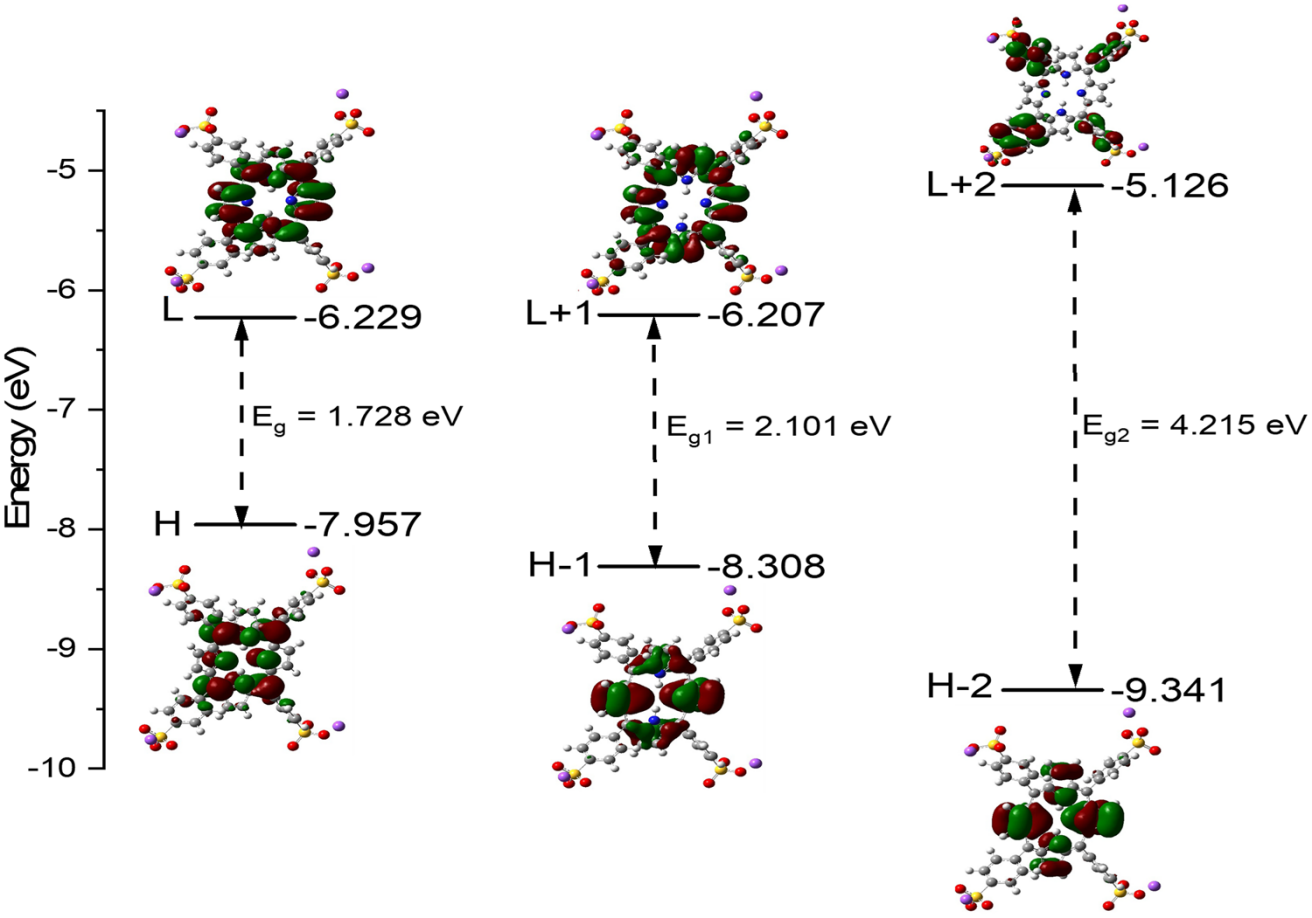
The MOs graphical representation of HOMO (H), LUMO (L), HOMO – 1 (H – 1), LUMO + 1 (L + 1), HOMO – 2 (H – 2), LUMO + 2 (L + 2), energy gaps between the following: H and L (E_g_), H -1 and L + 1 (E_g1_) and H -2 and L + 2 (E_g2_) (A) for TPPS GS

The molecular orbitals (MOs) graphical representation of the highest occupied molecular orbital (HOMO (H)), lowest unoccupied molecular orbital (LUMO (L)), HOMO – 1 (H – 1), LUMO + 1 (L + 1), HOMO – 2 (H – 2), LUMO + 2 (L + 2) and energy gaps between the following: H and L (E_g_), H -1 and L + 1 (E_g1_) and H -2 and L + 2 (E_g2_) for TPPS GS have been calculated: the results are collected in Fig. 7. As shown in Fig. 7, some important comments can be constructed as follow: (1) the H, L, H—1, L + l and H—2 MOs are localized over the PP group of TPPS GS. (2) H + 2 MO is localized over the four substituted phenyl groups. (3) The H and L MOs are π-bonding and π^*^-antibonding anti-bonding characters distributed over the whole target molecule. (4) Orbital energy level analysis and the resultant H – L energy gap (E_g_) referring to kinetic stability of a molecule [47]. A high E_g_ indicates high kinetic stability and low chemical reactivity because it is energetically unfavorable to add electrons to a high-lying L or to remove electrons from a low-lying H [47].

#### TD-DFT Calculations

**Fig. 8:**
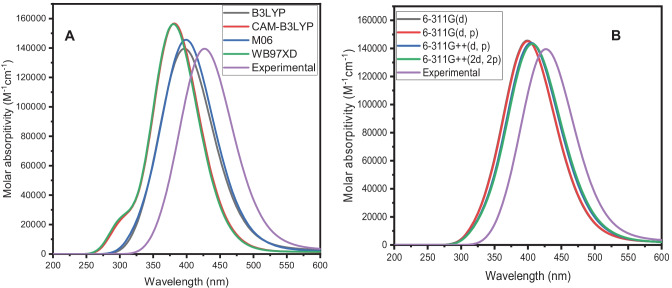
Calculated absorption spectra of TPPS GS obtained with the use of different functionals, and experimental absorption spectrum (**A**). The 6-311G(d) basis set was applied. Calculated absorption spectra of TPPS GS were obtained with the use of different basis sets, and experimental absorption spectrum (**B**). The M06 functional was applied

To achieve the optimum functional and basis set for calculating electronic absorption of the TPPS GS, various functionals, B3LYP [48], CAM-B3LYP [49], M06 [50], and wB97XD [51] were tested using 6-311G(d) basis set in water. The obtained resultant was collected in Fig. 8A in which M06 functionals give the nearest results to the experimental data of compound TPPS (See Fig. 8A). Also, to obtain the best basis set for calculating electronic absorption spectra for target compound, different basis sets, 6-311G(d), 6-311G (d, p), 6-311G +  + (d, p) and 6-311G +  + (2d, 2p) were test using M06 functional. The obtained calculated spectra were collected in Fig. 8B in which the 6-311G +  + (2d, 2p) basis set give the accurate results to the experimental one for target compound. The influence of applying various basis sets indicating calculated electronic absorption spectra of PPO GS is accurate with diffuse functions. Hence, involvement of diffuse functions is essential in obtaining accurate results. Therefore, the M06 functional and 6-311G +  + (2d, 2p) basis set is used to calculate the electronic absorption spectra for TPPS molecule in H_2_O solvent as presented in Fig. 8B.

The electronic absorption spectra of TPPS GS were obtained experimentally in water; the obtained electronic absorption spectra were presented in Fig. 8A and B. The experimental electronic absorption spectra of the target compound in an aqueous solution lie in the range (of 325—575 nm). This is owing to the π-π* transition. Also, the calculated electronic absorption spectra of TPPS compound using TD/M06/6-311G +  + (2d, 2p) in water lie in the range between (300 – 550 nm). Hence, the results data show that the computational absorption and experimental properties of the studied TPPS compound agree with the experimental data. The calculated excitation state, electronic transition, excitation energy (E_g_), oscillator strength (f), coefficient, and calculated maximum absorption wavelength (Th. λ_max_) parameters are listed in Table 3. The calculated electronic absorption spectra of TPPS GS in water appear as six transitions. The electronic transitions for TPPS at 591, 559, 411, 405, 362 and 363 nm as shown in Table 3. the third electronic transitions corresponding to the experimental peak at 411 nm (f = 1.571) arises from transition of MO258—> 262 (coefficient = 45%), MO260—> 263 (coefficient = 56%) and MO261—> 262 (coefficient = 33%) as presented in Table 3.

**Table 3:** Calculated electronic absorption such as (electronic state (ES), electronic transition (ET), excitation energy (EE), oscillator strength (f) and coefficient of TPPS GS, experimental absorption (Exp. λ_abs._ (nm)), and fluorescence ((Exp. λ_f_ (nm)) wavelength using TD/M06/6-311G +  + (2d, 2p)

TD-Computational						
ES	ET	E_g_ (eV)	f	Coefficient %	Th. λmax. (nm)	Exp. λmax. (nm)
1	260—> 263	2.097	0.020	36	591	324
	261—> 262			60		
2	260—> 26	2.215	0.025	40	559	
	261—> 263			56		
3	258—> 262	3.014	1.571	45	411	
	260—> 263			56		
	261—> 262			33		
4	260—> 262	3.057	1.898	56	405	
	261—> 263			40		
5	259—> 262	3.416	0.000	92	362	
6	258—> 262	3.588	0.487	87	363	

## Conclusion

The behavior of water-soluble TPPS absorption and fluorescence emission spectra has been studied in the presence of aqueous colloidal citrate capped Ag NPs and Au NPs. The observed UV–Vis absorption spectra of TPPS indicate the absence of interaction between TPPS with Ag NPs and Au NPs. But fluorescence quenching studies revealed a positive deviation from SV upon replacing Ag NPs with Au NPs. The latter behavior can be interpreted in terms of action sphere static quenching models. Various rate parameters for the fluorescence quenching process were determined by using a modified SV equation. The optimized TPPS GS using DFT/B3LY6-311G(d) was obtained successfully. The calculated electronic absorption spectra of TPPS GS in water appear as six transitions. The electronic transitions for TPPS at 591, 559, 411, 405, 362, and 363 nm. the third electronic transitions corresponding to the experimental peak at 411 nm (f = 1.571) arises from transition of MO258—> 262 (coefficient = 45%), MO260—> 263 (coefficient = 56%) and MO261—> 262 (coefficient = 33%).

## Data Availability

All data generated or analyzed during this study are included in this published article.
